# Technological Advances in Tumor-On-Chip Technology: From Bench to Bedside

**DOI:** 10.3390/cancers13164192

**Published:** 2021-08-20

**Authors:** Santa Bērziņa, Alexandra Harrison, Valérie Taly, Wenjin Xiao

**Affiliations:** Centre de Recherche des Cordeliers, INSERM, CNRS SNC 5096, Sorbonne Université, Université de Paris, 75006 Paris, France; santa.berzina@cri-paris.org (S.B.); alexandra.harrison@bristol.ac.uk (A.H.)

**Keywords:** tumor model, tumor-on-chip, on-chip analysis, cancer research, personalized medicine

## Abstract

**Simple Summary:**

Various 3D in vitro tumor models are rapidly advancing cancer research. Unlike animal models, they can be produced quickly and are amenable to high-throughput studies. Growing tumor spheroids in microfluidic tumor-on-chip platforms has particularly elevated the capabilities of such models. Tumor-on-chip devices can mimic multiple aspects of the dynamic in vivo tumor microenvironment in a precisely controlled manner. Moreover, new technologies for the on- and off-chip analysis of these tumor mimics are continuously emerging. There is thus an urgent need to review the latest developments in this rapidly progressing field. Here, we present an overview of the technological advances in tumor-on-chip technology by reviewing state-of-the-art tools for on-chip analysis. In particular, we evaluate the potential for tumor-on-chip technology to guide personalized cancer therapies. We strive to appeal to cancer researchers and biomedical engineers alike, informing on current progress, while provoking thought on the outstanding developments needed to achieve clinical-stage research.

**Abstract:**

Tumor-on-chip technology has cemented its importance as an in vitro tumor model for cancer research. Its ability to recapitulate different elements of the in vivo tumor microenvironment makes it promising for translational medicine, with potential application in enabling personalized anti-cancer therapies. Here, we provide an overview of the current technological advances for tumor-on-chip generation. To further elevate the functionalities of the technology, these approaches need to be coupled with effective analysis tools. This aspect of tumor-on-chip technology is often neglected in the current literature. We address this shortcoming by reviewing state-of-the-art on-chip analysis tools for microfluidic tumor models. Lastly, we focus on the current progress in tumor-on-chip devices using patient-derived samples and evaluate their potential for clinical research and personalized medicine applications.

## 1. Introduction

Tumors are complex heterogeneous structures composed of different cellular elements, including cancer, immune, stromal, and epithelial cells, as well as vascular components [1,2]. The tumor microenvironment (TME) encompasses cell–cell and cell–extracellular matrix (ECM) interplay between these components, in addition to mechanical pressures and chemical gradients. The complexity of the TME has made the development of physiologically representative tumor models an outstanding challenge (Table 1).

Animal tumor models have been accepted by regulatory agencies as preclinical platforms [3]. Nevertheless, animal-specific immune responses can limit the translational value of such models [1,2]. Although patient-derived tumor xenografts have demonstrated great potential for cancer research, they have shown different gene expression profiles than that of the original tumor [3,4], which makes them unsuitable for guiding tailored patient treatments. Additionally, animal models cannot offer high-throughput studies [5]; therefore, it is of utmost importance to develop sophisticated in vitro models that mimic the in vivo tumor by closely recapitulating various aspects of the TME. Moreover, these models should be simple to produce and able to generate results in a clinically relevant timeframe.

In the last decade, the trend in cancer research has shifted from the use of animal models towards the development of 3D in vitro tumor models. These models are able to reproduce the cell–cell and cell–ECM interactions in an environment that can reflect the 3D architecture of solid tumors, including the TME, unlike 2D cell monolayers. Technological developments in microfluidics have also consolidated their role in this field, in particular through the advent of tumor-on-chip platforms. Unlike other 3D in vitro models, such as tumor spheroids grown in suspension or on non-adherent surfaces, these platforms are able to mimic the dynamic properties of the TME, for instance by permitting the continuous perfusion of cell media for nutrient supply and waste removal. Moreover, in these systems, mechanical pressures and the chemical environment can be more readily and precisely controlled. Several reviews have covered tumor-on-chip development [6,7], as well as the achievements of in vitro mimicry of the TME [1,8]. In this review, we present a unique overview of the advances in tumor-on-chip technology by emphasizing state-of-the-art technologies for on-chip analysis. In particular, we evaluate the potential for tumor-on-chip technology to guide personalized cancer therapies by considering platforms that incorporate patient-derived samples.

## 2. Current Microfluidic Advances for Tumor-On-Chip Technology

In brief, tumor-on-chip denotes a microfluidic tumor model, which aims to maximize its physiological relevance by incorporating TME elements and accurately reflecting their in vivo properties and interplay. Establishing a standardized definition of “tumor-on-chip” is complicated by its differential use across papers, as well as an abundance of alternative terms. In this review, we classify any tumor spheroids developed and cultured on-chip as tumor-on-chip if they achieve higher-order TME mimicry in terms of cell–cell, cell–ECM or cell–immune interactions. This can be achieved through the co-culture of various cell line types, the culture of primary tumor spheroids or the engineering of vascularization. The use of dissociated patient-derived cells in tumor-on-chip devices is extensively covered in this review. We also consider the microfluidic culture of tumor slices from patient samples a form of tumor-on-chip technology.

Discussion of tumor-organoid-on-chip platforms is omitted from this review, which are distinguished from tumor-on-chip platforms by having a stem-cell-driven formation, using and retaining untransformed epithelial cells. Cancer organ chips elevate the tumor-on-chip concept by capturing organ-level physiology, rather than just the TME, and are reviewed elsewhere [9].

### 2.1. Progress in Tumor-On-Chip Technology

Many attempts have been made to develop physiologically relevant tumor-on-chip models for cancer research. Spheroids, as one of the most popular tumor-on-chip models, remain the gold standard and fill the need for simple in vitro tumor mimics. These models can mimic the organized cellular architecture present in solid tumors [10], although cell–cell interactions dominate over cell–matrix interactions [11]. Cancer cell lines can be individually cultured into spheroids, creating monocellular tumor spheroids. Nonetheless, these are unable to effectively mimic cellular interactions within the TME. To reach higher physiological relevance, cancer cell lines can be co-cultured with one another or with additional cell types to mimic intra- and intertumoral heterogeneity [1,10]. These multicellular tumor spheroids can be used to model different TME interactions, for instance by co-culturing tumor cells with fibroblasts, epithelial cells, or immune cells [2].

Tumors also have markedly different molecular and biological signatures from one another—a phenomenon known as intratumor heterogeneity [12]. Essentially, tumors are “malignant snowflakes’’ [13], which heavily affects drug efficacy, and consequently patient response to treatments. The best way to recapitulate this heterogeneity in vitro is through the use of patient-derived samples, including tumor slices, which can also effectively recapitulate the TME. Recently, personalized cancer treatments have attempted to tailor therapies to best match the unique tumor makeup of each patient [14,15]. Microfluidic platforms have the potential to enable this effort through both on-chip primary tumor spheroid generation and therapy testing. Moreover, microfluidics only require a small substance input [16]. This is particularly beneficial for personalized medicine, as the amount of patient derived samples can be extremely limited.

### 2.2. Technological Approaches for Tumor-On-Chip Technology

#### 2.2.1. Advances in Microfluidic Technologies

Although microfluidic approaches have been extensively implemented in various laboratories, the technology is still continuously being developed and improved. Microfluidic chips are frequently fabricated using soft lithography and photolithography-generated SU-8-patterned silicon wafer molds [11] with polydimethylsiloxane (PDMS) as the principal material [17,18]. Although PDMS is an elastic, chemically inert, transparent, biocompatible [19] and gas permeable [2,20] material, it has been reported to absorb small molecules [21]. This characteristic makes PDMS undesirable for drug testing, as the actual drug concentrations within the microfluidic device may vary from the theoretical values [8,21,22]. Moreover, even if gas permeability of PDMS is often presented as an advantage, it could also be a disadvantage, for example when hypoxic conditions are preferred.

Therefore, alternative materials for microfluidic device fabrication have been implemented and developed. A longstanding technological approach involves using glass or glass-silicon fabricated devices. The hydrophilicity of such devices ensures little absorption of proteins, drugs, and metabolites [23,24]. Furthermore, agarose is known to be non-adhesive and suitable for spheroid formation by cell–cell interactions [25]. Also poly(methyl methacrylate) (PMMA) is less drug-absorbent than PDMS [21] and is an oxygen-impermeable material [20]. Consequently, it has been used in microfluidic chip fabrication to block oxygen diffusion [20]. Palacio-Castañeda and colleagues developed a PDMS chip covered by a 175-µm-thick PMMA film. The resulting structure reportedly maintained hypoxic conditions in the microchannels. Moreover, it was optically transparent, making it suitable for external optical interrogation [20]. Michael and coworkers developed a hanging drop paper microfluidic device by means of paper surface engineering [26]. To ensure its hydrophobicity, a wax printer was used to imprint a pattern on qualitative filter paper. The resulting object was heated and treated with bovine serum albumin (BSA) to form hydrophilic wells, demonstrating a simple, easy to operate, and inexpensive microfluidic platform. Their chip is compatible with 3D tumor model generation, demonstrated through the formation of tumor spheroids from co-cultured breast cancer cells and skin fibroblasts.

Stereolithography focused 3D printing technologies have been used as alternatives to traditional photolithography for wafer and mold production [25,27]. Although photolithography is able to produce structures in high resolution of nanometer scale [28], 3D printing allows more versatile device design and increases the biocompatibility of the system. Furthermore, it removes the need for manual post-processing at a lower cost; therefore, 3D printing technologies might be more desirable and accessible for microfluidic platform development [29].

**Figure 1:**
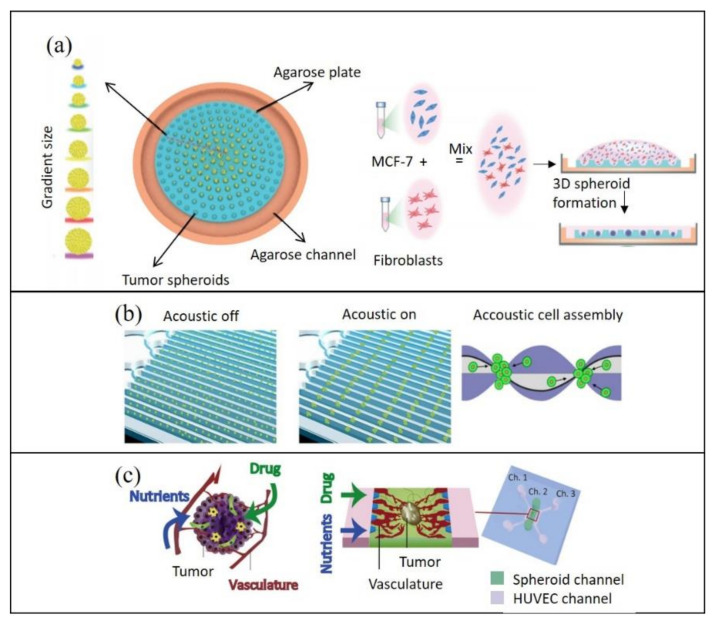
Representative schematics of technological advances in microfluidics for tumor-on-chip generation: (**a**) an agarose microfluidic chip capable of position-dependent distribution of tumor spheroids, with gradient-controlled sizes under a liquid dome (left) and co-culture of a breast cancer cell line (MCF-7) and human dermal fibroblasts (right) (adapted from [25]); (**b**) an acoustofluidic high-throughput tumor spheroid assembly platform with multiple channels and a reusable surface acoustic wave generator (left), also showing the tumor spheroid formation (right) (adapted from [11]); (**c**) vasculature of an in vivo tumor (left) and recapitulation of the vascular network for in vitro microfluidic tumor models (right) (adapted from [30]).

Additionally, 3D-printed resin molds have also been used to develop microfluidic devices using PDMS alternatives, such as agarose [25,27]. Fang and co-workers successfully demonstrated tumor spheroid development on a 3D-printed agarose microfluidic chip [25]. The tri-culture of MCF7, human dermal fibroblasts and HUVECs resulted in the formation of differently sized MCTS across a single chip under a liquid dome. The platform additionally served as an efficient means for drug testing, allowing the simultaneous assessment of drug efficacy for variously sized spheroids (Figure 1a).

Recent advances in 3D printing have resulted in higher resolution and have unlocked the possibility of direct microfluidic platform fabrication instead of mold printing [17,31]. Liquid resins, such as Pro3dure GR-10 and MED 610, have been used for 3D-printed microfluidic platform fabrication [32]. These materials have shown chemical inertness as well as a reduced tendency to absorb small molecules in comparison to PDMS. They also demonstrated comparable biocompatibility with human T-lymphocytes [32] and immunotherapeutic agent response assessment on biopsied tumor tissue [17]. Nonetheless, such materials often suffer from a lack of transparency, limiting their compatibility with external optical analysis [17,31]. There have been different attempts at making 3D-printed microfluidic devices more transparent, such as sanding both sides and then further post-curing [33]. Alternatively, Bazaz and colleagues attached transparent PMMA sheets to their 3D-printed microchannels, permitting optical and fluorescent microscopy [34]. Finally, this issue may be further addressed in the future with the ongoing development and optimization of transparent resins or resins with strong optical properties [35,36,37]. We therefore believe that 3D printing is likely to be used increasingly in tumor-on-chip microfluidic platform fabrication to address the current shortcomings of PDMS.

#### 2.2.2. Reconstituting Cell–Cell Interactions On-Chip

In tumor models, cell–cell interactions, particularly heterogeneous interactions, are required to recapitulate in vivo paracrine signaling between the different cell types of the TME [38]. As mentioned previously, these cell–cell interactions can be readily established in multicellular tumor spheroids. Jeong and co-workers showed that the growth of HT-29 human colorectal carcinoma cells in tumor spheroids was stimulated upon their co-culture with fibroblasts, demonstrating the influence of cell–cell interactions within in vitro tumor models [39]. Cell–cell interactions are also important for recapitulation of the epithelial-to-mesenchymal transition, which plays a crucial role in cancer progression and metastasis [40].

One standard approach to developing multicellular tumor spheroids is through hanging drop culture on a microfluidic platform, which promotes spheroid formation following gravity [18,26,41]. Droplet microfluidic platforms have been developed to facilitate the generation of multicellular tumor spheroids by mixing and encapsulating cells in a single droplet, which is protected by an immiscible liquid phase [42,43,44]. Taking advantage of a high surface-area-to-volume ratio, these platforms offer the possibility of high-throughput and homogeneous spheroid formation [45].

More recently, new tissue engineering and cell–cell interaction modeling approaches have emerged as further alternatives. For instance, a high-throughput, proof-of-concept spheroid formation approach using acoustofluidics has been reported (Figure 1b). Chen and co-workers used a standing surface acoustic wave generator to form pressure nodes in the microfluidic device and successfully created tumor cell aggregates. Upon introduction in a microfluidic channel, cells moved towards pressure nodes to assemble into aggregates, which could be matured to form strong cell–cell interactions [11]. Alhasan and colleagues have similarly coupled a microfluidic surface acoustic wave generator to the microwell of a tissue culture plate. Although spheroids were formed off-chip in the microwell, they managed to do so in a quicker time frame than what is typically needed for the hanging drop method [46]; thus, acoustofluidic technologies may be a valuable tool to facilitate in vitro spheroid formation for cancer research.

Finally, although still in its infancy, 3D bioprinting may emerge as an advantageous method of microfluidic spheroid generation. For instance, Bhise and colleagues have successfully bioprinted a mix of pre-formed HepG2/C3A spheroids with GelMA as liquid droplets in a bioreactor [47].

#### 2.2.3. Reconstituting the Biochemical Microenvironment On-Chip

Solid tumors have a multi-layered structure, with each layer having a unique TME composition in terms of its pH, nutrient/oxygen distribution, and metabolic waste products [25,48]. These are challenging to reconstitute in vitro, as microfluidic platforms are generally designed to use small sample volumes and grow spheroids within a confined space. To address these challenges, the recapitulation of the vascularization in microfluidics has thus been proposed for accurate tumor model development [30,49]. The vascular network ensures the establishment of multiple in vivo chemical gradients [16] and is of particular interest to recapitulate in vitro.

Although co-culturing cancer cells with endothelial cells has been suggested to allow the process of vascularization [30], other TME interactions also play a pivotal role in this process. For example, Nashimoto and colleagues successfully developed vascularized tri-culture tumor spheroids consisting of human umbilical vein endothelial cells (HUVECs), human lung fibroblasts (hLFs) and a human breast cancer cell line (MCF-7) [30]. Interestingly, successful formation of one large blood vessel was observed in the center of the spheroids, consisting of HUVECs and surrounded by hLFs, which provided a chemical gradient (Figure 1c). Despite its complexity, recent advances have been made towards the development of perfusable vascularized networks in tumor-on-chip technology [30,50,51]. For instance, vascularization can be achieved with bioprinted blood and lymphatic vessels [52]. Furthermore, the subjection of tumor cells to hypoxic conditions has also been reported to stimulate angiogenesis—the first step to vascular network formation [53].

The modeling of different oxygen levels is also essential for on-chip TME reconstitution, as normoxic, hypoxic and almost fully anoxic regions are found in cancerous tissues [20]. To achieve this, Grist and colleagues developed a microfluidic platform, in which breast tumor cell (MCF-7) spheroids in alginate hydrogel core–shell beads were cultured. All tumor spheroids were periodically exposed to 0%, 3% and 10% oxygen [54]. They discovered oxygen profile-dependent swelling and shrinking of individual cells within the tumor spheroids. Moreover, these different oxygen levels can influence cellular responses, for instance to doxorubicin treatments. Palacio-Castañeda and colleagues also demonstrated the generation and maintenance of hypoxic conditions by firstly establishing an anoxic environment on-chip and then monitoring oxygen diffusion through their device under normal atmospheric conditions [20]. We believe that both models have succeeded in elevating the physiological relevance of tumor-on-chip platforms.

Cell–ECM interactions further contribute to the tumor microenvironment, with the ECM having been shown to enhance the growth of cancer cells in vitro [53]. To reconstitute the ECM on-chip, synthetic scaffold-based culturing methods are frequently chosen, because they focus on culturing cell types in a 3D environment that is able to closely mimic natural ECM. Scaffolds can be synthetic, such as poly(ethylene glycol) [55], or naturally derived hydrogel matrices, including Matrigel or collagen I and IV [8,56]. Although these polymers are similar to the ECM components found in solid tumors, their characteristics vary in terms of composition, structure and stiffness, depending on their method of production [6]. For example, a dense ECM microstructure can result in increased fluid pressure within the device, which could result in cell damage and further influence drug responses and tumor resistance [57]. Moreover, a recent tendency in ECM modeling in vitro involves matrix stiffness mimicry [38,56], as it greatly contributes to the tumor phenotype [58]; therefore, we believe that successful tumor matrix mimicry would be a major step for tumor-on-chip development.

## 3. Current Advances in Tumor-On-Chip Analyses

While new microfluidic tumor-on-chip platforms are continuously being developed for 3D tumor modeling, the utility of these devices is dependent upon techniques for effective analysis of the resulting tumor models. Only in this manner can biological information be gleaned for impactful translational research, including drug testing. Several techniques have, thus, been developed or applied for the analysis of 3D tumor models [59], yet the advent of microfluidic tumor model generation necessitates the concomitant development of tools compatible with or optimized for these platforms. Despite their undeniable importance, tools and techniques for on-chip analysis are, to the authors’ knowledge, an area frequently missing in current tumor-on-chip and microfluidic tumor spheroid reviews. To address this, we herein provide an overview of the state-of-the-art technologies for microfluidic tumor spheroid and TME analysis, including those that can be integrated with the microfluidic platform. We emphasize the translational potential of their readouts—in particular for drug testing.

### 3.1. Physical Sensors On-Chip

In a pioneering work by Shrike Zhang and colleagues, physical and biochemical sensors were integrated into an organ-on-chip platform to monitor the microenvironment in situ [22]. Integrating similar analysis methods on-chip to assess established tumor models is highly desirable. The implementation of physical sensors on-chip for the evaluation of the TME—for instance in terms of pH, oxygen, and temperature—has been reported [22]. Physical sensing methods do not involve any labeling procedures and are advantageous for molecular absorption, distribution, metabolism, and excretion (ADME) studies. Essentially, the chemical ions within the fluid system can transmit current and can be correlated with pH, spheroid size and viability. For example, electrochemical sensors have been successfully used for the measurement of glucose and lactate levels based on electron transfer during oxidation–reduction reactions. These assisted in determining the metabolic behavior of tumor microtissues derived from a colorectal cancer cell line HCT116, particularly the growth and viability of the cells [18]. By the same token, miniaturized electrochemical pH sensors have been implemented for on-chip pH sensing to avoid additional chemical input [60]. Impedance spectroscopy has also been reported as a viable droplet microfluidic tool for measuring tumor spheroid size on-chip, by detecting voltage in different frequencies while applying a constant current to the droplets [41]. In a study conducted by Wu and colleagues, impedance spectroscopy was used to monitor tumor spheroid viability throughout drug testing assays [61]. When the spheroid viability and morphology change, the cell adherence to electrodes is affected, which leads to changes in impedance values. This study demonstrates how on-chip integrated analytical tools, such as physical sensors, have the potential to be applied to tumor-on-chip treatment evaluation. They are particularly likely to play an important role in the field, given the rising development of tumor-on-chip devices for personalized medicine applications.

### 3.2. On-Chip Imaging

Optical imaging is crucial for the characterization of tumor models, particularly in terms of size, morphology, viability and molecular composition via protein or metabolite detection. Confocal laser scanning microscopy (CLSM) is widely used for these intents. Nonetheless, photon scattering decreases signal strength with imaging depth and limits the capability of CLSM for high-resolution imaging of deep tissue layers [62]. Other modalities such as light-sheet-based fluorescence microscopy [62] and multi-photon microscopy afford better specimen penetration (up to 500–800 μm) and resolution at inner cell layers [63]. Nonetheless, these techniques are expensive and laborious, often requiring specialized personnel; therefore, there is a high demand for new imaging technologies that enable deep-tissue imaging of microfluidic tumor models with high throughput and low cost.

**Figure 2:**
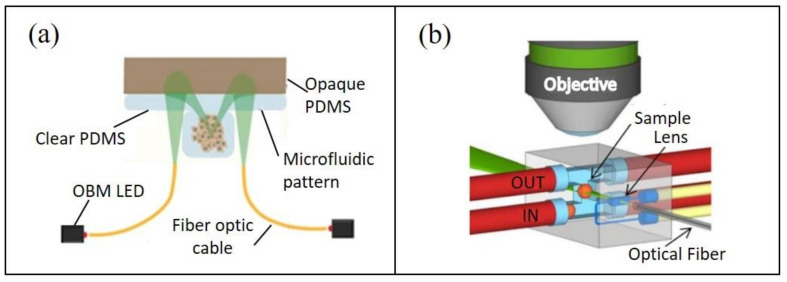
Microfluidic tumor imaging techniques: (**a**) schematic of the oblique back-scattered illumination pathway of the Spheroscope for monitoring microfluidic tumor spheroid growth (adapted from [66]); (**b**) schematic of a device for selective plane illumination microscopy on-chip, using an optofluidic approach (adapted from [68]).

St Georges-Robillard and colleagues used wide-field fluorescence hyperspectral imaging (HSI) for high-throughput analysis of ovarian tumor spheroids in microfluidic chips [64]. While having a lower spatial resolution than confocal imaging, this technique enabled up to 60 spheroids to be captured in a single acquisition with increased light penetration depth. They demonstrated its use as a tool for both preformed and on-chip-generated spheroid analysis, and further commented on its compatibility with ex vivo tumor-on-chip models [65]. In this manner, spheroid growth can be studied rapidly and in a non-destructive manner at multiple time points, with the potential to accelerate studies on spheroid response to molecular agents. Rodríguez-Pena and colleagues argue that current microscopy systems used for spheroid growth analysis are overly complex and expensive for this relatively simple objective [66]. To address this incongruity, they developed a miniaturized, inverted microscope composed of low-cost parts (Figure 2a). Using this device, fluorescent and phase-gradient images were acquired to track lung cancer cell line spheroid formation and growth in a microfluidic chip. Using this “Spheroscope” could, thus, improve the affordability of spheroid drug response studies, permitting increased experimental flexibility and potential to scale up for high-throughput studies. Another approach to facilitate portable and low-cost 3D imaging of tumor tissues involves the integration of optics with microfluidics— “optofluidics”. Here, optical elements can be incorporated into the microfluidic chip for increased automation and portability [67] (Figure 2b). In the context of 3D tumor model imaging, it is the authors’ understanding that optofluidics have only been implemented as a preformed tumor spheroid analysis tool [68]. In the future, we expect this field to elevate tumor-on-chip automation and imaging capabilities.

Grist and colleagues have integrated tissue clearing protocols on a microfluidic platform to address the need for deep tissue imaging tools compatible with microfluidic chips [69]. Tissue clearing renders thick biological samples transparent, allowing for their effective, deep layer imaging without sectioning. Following clearing, tumor spheroids of breast cancer cells (MCF-7) embedded within hydrogel beads were fluorescently imaged with increased penetration depth. Furthermore, the compatibility of their device with on-chip 3D cell culture holds promise for its eventual progression into a tumor-on-chip platform for image-based cancer drug testing. Other microfluidic devices exist for spheroid clearing [70,71], although to the authors’ knowledge, do not currently specify compatibility with tumor spheroid generation and culture. Nonetheless, tissue clearing could induce artefacts of structure or tissue alterations and is typically only suitable for endpoint analyses.

In addition to on-chip imaging technologies, advances in image analysis are necessary in order to effectively manage the data—especially high-throughput data. Computational tools using machine learning, such as image pattern recognition algorithms, have been implemented to effectively analyze microfluidic tumors [72]. For example, the HSI imaging system described earlier can perform clonogenic assays and quantify tumor spheroid composition [65]. This was achieved through image analysis on hyperspectral data cubes, in which regions of interest were selected for each spheroid and the spheroid cellular composition was calculated. This obviates the need for destructive, off-chip spheroid composition analyses such as flow cytometry. Furthermore, it has been proposed that machine learning algorithms and artificial intelligence will become increasingly integrated into tumor-on-chip platforms for big data processing [73]. As an example, neural networks have been trained to be able to estimate spheroid viability from brightfield images, eliminating the need for traditional live–dead staining and fluorescence imaging [74]. This approach was used to estimate the efficacy of various chemotherapy drugs on triple-negative breast cancer cell (SUM159) tumor spheroids grown on a microfluidic chip. Nonetheless, the integration of machine learning algorithms for microfluidic tumor model analysis remains in its infancy, with few demonstrated examples.

The given examples focus on imaging technologies for spheroid growth tracking and viability assays. Nonetheless, the capabilities of microfluidic technologies can also be coupled with imaging tools to unravel the molecular behavior of spheroids. As an example, Saint-Sardos and colleagues recently developed their droplet microfluidic platform to enable the measurement of both intracellular and secreted cytokines from spheroids [45]. A secondary droplet, containing anti-VEGF-A beads and anti-VEGF-A secondary fluorescent antibody, was delivered and anchored next to the spheroid droplets. In this manner, the level of VEGF-A secretion from the spheroids could be quantified by fluorescence microscopy. Although this technology remains to be tested on tumor spheroids, it has the potential to aid our understanding of intratumor cell–cell interactions and their role in drug resistance.

### 3.3. Tumor Recovery for Off-Chip Analysis

The capability of tumor recovery for downstream analysis has recently been described as “conclusively essential” for in vitro cultures [75]. By extracting intact tumor models and preserving their 3D structure, a range of invasive, off-chip analyses can be performed, including examining histology and microdissection. Although the inability to extract spheroids is argued to be a major limitation of many tumor-on-chip systems [76], several techniques have been developed to allow simple and effective tumor model recovery from microfluidic platforms.

**Figure 3:**
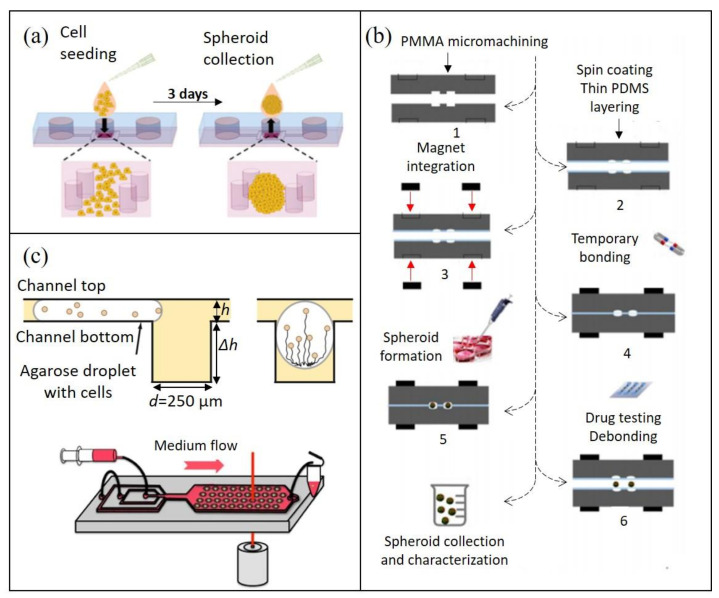
Tumor spheroid recovery for off-chip analysis: (**a**) the process of cell seeding and spheroid harvesting by pipetting from a microfluidic pillar array device (adapted from [78]); (**b**) schematic of the fabrication of a reversibly sealed microfluidic device and its subsequent use for tumor microtissue cultivation, drug testing and recovery (adapted from [81]); (**c**) schematic of spheroid formation within an agarose droplet after trapping and sedimentation into an anchor on the microfluidic chip (top), as well as controlled agarose embedded spheroid recovery from a microfluidic device by selective heating with an infrared laser beam (bottom) (adapted from [42]).

Microfluidic platforms can be designed to simplify tumor model recovery. For instance, open-format microfluidic devices can permit direct access to and recovery of generated tumor spheroids by simple pipetting (Figure 3a) [77,78]. Dismantable microfluidic devices can also permit top microchannels to be peeled off, facilitating tumor spheroid retrieval from the device by pipetting [79]. Similarly, Ayuso and colleagues designed a microdevice, whose upper half can be removed for the collection of collagen hydrogel-embedded tumor cells using a biopsy punch [80]. Elevating this concept even further, microfluidic devices can be reversibly sealed, for instance by means of an integrated magnetic system (Figure 3b) [81]. Through this, spherical microtissues generated from a colorectal cancer cell line HT-29 were effectively recovered from the microwells. Ultrahigh-resolution scanning electron microscopy was then used to investigate the effects of drug treatment on the morphology of the spheroid surface. This illustrates how off-chip characterization, following proper tumor recovery, has the potential to enhance drug testing studies. Other microfluidic devices have also demonstrated successful spheroid recovery by using PCR adhesive tape [82] or spring-loading force [83] to reversibly seal the microfluidic chips. Recovered tumor spheroids can also be dissociated for subsequent single-cell analyses. For instance, the aforementioned device of Zhao enabled individual gene expression profiling of the dissociated spheroids [79]. Moreover, Patra and colleagues used a “peelable” microfluidic device to study the combined effects of radiotherapy and doxorubicin treatment on soft tissue sarcoma spheroids, applying flow cytometry and clonogenic assays on the dissociated spheroids post-treatment [84]. The authors emphasize their workflow’s potential for drug discovery and the evaluation of optimal radiotherapy–chemotherapy synergies—a combination often used in clinical treatments.

In closed-format tumor-on-chip systems, the reversal of the flow direction or increase in flow rates is typically used for spheroid recovery [10,85]. Such an approach is likely to retrieve all the spheroids on a chip, rather than particular ones of interest. To address this, Sart and colleagues built a droplet microfluidic platform, which uses integrated anchors to trap the droplets. Spheroids were formed by subsequent cell sedimentation at the bottom of the droplets (Figure 3c, Top). After the treatment, spheroids were recovered by using a focused infrared laser followed by culture media flow to collect agarose-embedded tumor spheroids [42] (Figure 3c, Bottom). In this manner, the heat of the laser selectively detaches spheroids, which are subsequently flushed out of the device. The retrieval of particular spheroids over bulk is advantageous when only a subset of spheroids is of interest to the analysis, alleviating the need for post-recovery sorting. The argument has been raised that the shear stress demanded by such flowrate controlling methods could be cell damaging [81]. Nonetheless, several studies have reported the culture of viable spheroids up to 2 weeks post-recovery [85], great spheroid integrity [10] and continued cell migration capabilities [42].

## 4. Applications and Clinical Aspects of Tumor-On-Chip Technology

### 4.1. Potential Applications of Tumor-On-Chip Technology in Personalized Medicine

As previously outlined, tumor heterogeneity limits the success of a “one-size-fits-all” treatment approach to cancer; therefore, extensive efforts have been made towards the effective tailoring of cancer therapies for individuals or stratified groups of patients. While next generation sequencing (NGS) can be used to guide such approaches [86], ex vivo drug testing on solid patient tumor samples and biopsies has emerged as a complementary tool, with the potential to more precisely determine optimal therapy regimens. To this end, several teams have been developing tumor-on-chip platforms that support the culture of patient-derived tumor samples and evaluation of their response to therapies. A comprehensive overview of recent progress made in this field is provided in Table 2.

**Figure 4:**
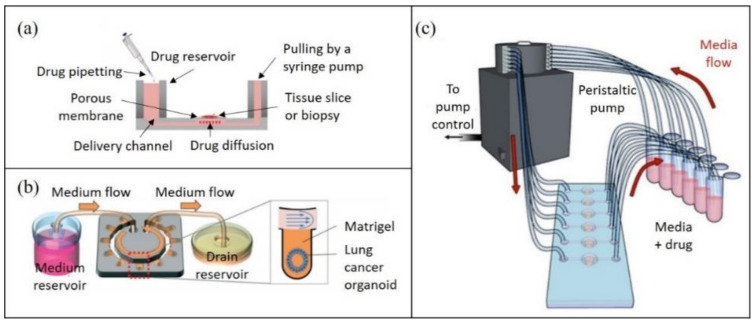
Representative tumor-on-chip platforms for potential applications in personalized medicine: (**a**) cross-sectional representation of a tumor tissue slice cultured on a porous membrane (adapted from [21]); (**b**) cartoon depiction of a microfluidic yarn flow resistor passive microflow device for human lung organoid development and culture (adapted from [89]); (**c**) schematic depiction of a microfluidic device plug-in loop to enable cell media delivery to cultured spheroids from a reservoir using a peristaltic pump (adapted from [90]).

Tumor tissue typically undergoes physical mincing and enzymatic digestion into a single-cell suspension before on-chip culture. These patient-derived cells can be seeded onto the microfluidic device shortly after dissociation [87] or may first undergo several passages in off-chip cultures [88], and occasionally off-chip spheroid preformation [31]. Alternatively, tumor slices or microdissected tumor tissue can be cultured on tumor-on-chip platforms [15,21]. For example, Rodriguez and colleagues developed a PMMA microfluidic device capable of culturing up to 40 patient-derived tumor tissue samples for drug screening (Figure 4a) [21]. Alternatively, patient biopsies can be cultured into organoids on a microfluidic platform, as recently demonstrated for lung cancer organoids (Figure 4b) [89]. On this platform, the patient-derived organoids were subsequently subjected to drug screening, using a continuous flow of drug-containing media to model physiologically relevant flow conditions.

Most tumor-on-chip platforms using patient-derived samples have focused on chemotherapy drug testing. For example, by using dissociated tumor biopsies from mesothelioma patients, Mazzocchi and colleagues demonstrated long-term culture of viable 3D spheroids within 6 chambers, with subsequent chemotherapeutic drug testing [90]. A peristaltic pump was used to facilitate the continuous flow of media or drug-infused media to the spheroids (Figure 4c). Crucially, the drug responses of patients were recapitulated in the on-chip tumor spheroids, strongly supporting the utility of this approach for precision medicine approaches. Lim and colleagues cultured spheroids of biopsy-derived primary breast cancer cells in a size-controllable manner, treating them with the same chemotherapy drugs as those administered to the patients [78]. They too reported similarities in the drug response of their spheroids as witnessed for the patients. Therein, these two examples, along with the work of the other teams provided in Table 2, emphasize the encouraging progress made with tumor-on-chip technology in regard to tailoring chemotherapy treatments.

More recently, several groups have also started investigating the use of tumor-on-chip technology for the evaluation of combination therapies. For example, a droplet microfluidic platform was engineered to permit the generation of chemically distinct droplets [14]. This was used to conduct pairwise drug testing on patient-derived pancreatic tumor cells, investigating the effects of chemo-, targeted and cytokine combined therapies. The same group has also demonstrated effective coupling of such microfluidic-based perturbation screenings with mathematical logic models, using established knowledge of signaling pathways to unravel patient-specific signaling pathways, which in turn can then be used to guide personalized therapies [91]. Implementing in silico testing also allows prediction of the effects of new potential therapies that cannot be tested experimentally due to the limited amount of solid tumor or biopsy material.

Other groups have explored the possibility of using tumor-on-chip technology for the personalization of radio- and immunotherapy. The associated comorbidities of radiotherapy justify the development of a tool to predict patient radiosensitivity and to assess the value of treatment. With this in mind, precision cut head and neck cancer tumor slices were cultured and irradiated on a tumor-on-chip device [15]. Their easy-to-use polyetheretherketone (PEEK) microfluidic device was capable of maintaining tissue viability for 68 h, and an irradiation system enabled precise radiotherapy treatment delivery. With regards to immunotherapy, Aref and colleagues developed patient-derived organotypic tumor spheroids (PDOTS) on-chip to test responses to immune checkpoint blockade using antibody treatment [92]. Since there are currently no known reliable genetic signatures or biomarkers for prediction of immunotherapy resistance, this platform represents a novel approach to understand and overcome this issue.

### 4.2. Clinical Perspective of Tumor-On-Chip Technology

The rise in microfluidic technologies and analysis tools described in this review has undoubtedly enhanced the multifunctionality of tumor-on-chip platforms. They are, thus, likely to facilitate the use of tumor-on-chip technology in personalized medicine, which to the authors’ knowledge, has not yet advanced to clinical-stage research or applications. Nonetheless, the described proof-of-concept tools have insofar largely been applied on tumor spheroids, with most examples having yet to be successfully integrated with more sophisticated tumor models, especially using patient-derived samples. Furthermore, the integration of certain analysis tools inro tumor-on-chip devices could inadvertently complicate their production and operation, limiting the reproducibility of the data. In order to transition from bench to bedside, we believe that an optimal trade-off needs to be made between the technological complexity and simplicity of tumor-on-chip devices, as well as affordability of both their manufacture and operation. Furthermore, most of the cutting-edge technologies discussed in this review need further optimization and lower costs in order to be implemented in the broader scientific community. Implementation would also require specifically trained staff, which is time-consuming. The rise in 3D printing technologies is likely to alleviate these challenges by enabling direct printing and the use of PDMS alternatives, as described earlier. Additionally, 3D printing can afford shorter fabrication times and greater affordability than traditional methods [17]. Further automatization of tumor-on-chip production is also likely to ensure simple scale-up and adoption in clinical settings. To make operation more economically feasible, tumor-on-chip devices would also benefit from higher throughput and parallelization. Notwithstanding, Eduati and colleagues developed a platform that enables the workflow of drug testing on live patient cells to be completed within 48 h for only 150 USD per patient [14].

Another major challenge for testing the therapeutic response of patient-derived samples is their small quantity and short lifetime in ex vivo cultures. To address these, tumor-on-chip devices must be compatible with the processing and analysis of small amounts of tumor specimens within a clinically relevant timeframe. Bower and colleagues succeeded in dissociating and culturing fresh tissue samples on a microfluidic platform within 90 min of excision [87]. Nonetheless, the use of freshly resected tumor cells can result in poor viability on-chip, necessitating the culturing of cells outside the chips prior to seeding [88]. Such steps may reduce the physiological relevance of the final tumor-on-chip platform. In particular, it has been argued that methods using patient-derived cells from dissociated tumor tissue typically lack the TME that is present in vivo [21]. Several groups have demonstrated the successful maintenance of tumor slices on microfluidic chips for over 72 h [90,99], which obviates the need for extensive ex vivo processing or culturing steps, making them an alternative suited for future clinical applications.

Finally, the reflection of the laboratory results in real cases has only been reported for a limited number of patients [78,90]. This is likely due to difficulties associated with obtaining patient samples, metadata and patient outcome data [100]. As such, it is difficult to evaluate the ability of these platforms to accurately reflect patient-specific therapy responses in a reproducible manner. The lack of standardization of tumor-on-chip fabrication and use also limits the extent to which data can be compared and corroborated across laboratories. Nonetheless, we believe that the clinical correlation of laboratory results is likely to be met once the devices enter clinical stage research. We further emphasize that the establishment of consortia or collaborations between laboratories, biobanks, clinics and the biotechnology or pharmaceutical industry will facilitate this transition. International initiatives for personalized cancer medicine have been created, such as the WIN consortium. Although the focal point continues to be genomic and transcriptomic approaches to precision oncology [101], such initiatives are likely to pave the way for clinical tumor-on-chip implementation in the future.

## 5. Conclusions

Clinically applicable tumor-on-chip platforms should be high-throughput, simple, reliable, easy-to-use, and cheap to produce and operate. Furthermore, they should maintain sample viability, as well as mimic the TME and patient therapy responses. We believe that the reviewed tumor-on-chip platforms meet several of these requirements but still struggle to satisfy them all. In particular, clinical corroboration of the laboratory results is still needed to ascertain that patient-specific therapy responses are reflected in these platforms. Accurate recapitulation of the TME on-chip can be highly beneficial in ensuring such corroborative results. Moreover, the rise in 3D printing technologies is likely to enable quick and low-cost production of complex tumor-on-chip devices. Finally, new and optimized on-chip analysis techniques are enhancing the sophistication and multifunctionality of tumor-on-chip devices. These techniques are likely to catalyze on the implementation of tumor-on-chip devices clinical settings, provided they do not compromise the simplicity of manufacture and operation. Overall, we believe that these technological advances in tumor-on-chip devices are paving the way for an exciting new age of personalized cancer medicine.

## Figures and Tables

**Table 1:** Advantages and disadvantages of different tumor models.

Type of Tumor Model	In Vivo/In Vitro/Ex Vivo	Dimension	Recapitulate ECM and Cell–Cell Interactions	Recapitulate TME Dynamics	Translational Value	Advantages	Disadvantages
Cancer cell line monolayers	in vitro	2D	No	No	Low	Inexpensive Not labor-intensive and requires limited expertise High-throughput possible	Limited recapitulation of in vivo environment and subsequent translational value
Primary cancer cell monolayers	in vitro	2D	No	No	Low	Inexpensive Not labor-intensive and requires limited expertise High-throughput possible Less artificial than cancer cell line monolayers	Limited recapitulation of in vivo environment and subsequent translational value
Cell line monocellular tumor spheroids	in vitro	3D	No	No	Low	Inexpensive High-throughput possible	Difficult to maintain uniformity of spheroid size
Cell line multicellular tumor spheroids	in vitro	3D	Yes	No	Low	Inexpensive High-throughput possible	Difficult to maintain uniformity of spheroid size
Microfluidic cell line multicellular tumor spheroids	in vitro	3D	Yes	Yes	Intermediate	Can model chemical gradients High-throughput possible	More technically challenging than 2D models and cell line tumor spheroids
Microfluidic patient-derived tumor spheroids	ex vivo	3D	Yes	Yes	High	May contain natural ECM Cheaper than animal models High-throughput possible	More technically challenging than cell line models May genetically vary from parent tumors Difficult to access patient tumor tissue Difficult to maintain uniformity of spheroid size
Microfluidic patient-derived tumor tissue	ex vivo	3D	Yes	Yes	High	May contain natural ECM Cheaper than animal models High-throughput possible	More technically challenging than cell line models May genetically vary from parent tumors Difficult to access patient tumor tissue Difficult to maintain uniformity of spheroid size
Patient-derived tumor spheroids	ex vivo	3D	Yes	No	Intermediate	Cheaper than animal models May contain natural ECM High-throughput possible	Difficult to maintain uniformity of spheroid size May genetically vary from parent tumors
Tumor tissue slice/explant culture	ex vivo	3D	Yes	Yes	High	Cheaper than animal models Contain natural ECM High-throughput possible	Not easily amenable for high-throughput studies Limited amount of sample Difficult to access patient tumor tissue
Animal tumors	in vivo	3D	Yes	Yes	Intermediate	Accepted by agencies as a pre-clinical platform Testing within a living organism accounts for interactions between organs	Species specific differences to treatments [1] Specific facilities and highly trained staff required Ethical approval needed Low throughput Expensive and time-consuming
PDX Tumors	in vivo	3D	Yes	Yes	High	Accepted by agencies as a pre-clinical platform Testing within a living organism accounts for interactions between organs	Limited amount of samples Animal-specific immune responses limit fidelity to patient responses [3] Transcriptome may vary from that of the original tumor [4] Lack of standardization Requires specific facilities and highly trained staff Ethical approval needed Not easily amenable for high-throughput studies Expensive and time-consuming

**Table 2:** Tumor-on-chip models incorporating patient-derived samples for therapy testing.

Year	Sample Type	Cell Culture	Microfluidic Device	Application	Analysis	Main Outcomes	Ref.
	Chemotherapy						
2015	Primary human lung tumors and squamous carcinoma tissues	Dissociated cells from tissues; monocultured or co-cultured	Two PDMS parts consisting of microwells, fabricated by stereolithography epoxy molds, plasma-bonded	Drug treatment with cisplatin for 48 h	**On-chip:** Live–dead staining and fluorescence imaging to assess cell viability **Off-chip:** Flow cytometry for cell sorting, followed by caspase-3/7 activity of the supernatant to assess apoptosis	System efficiency was high with little cell loss in the microfluidic network. Primary pericytes (PCs) had a protective effect on the primary epithelial lung tumor cells (PLETCs) from the damaging effects of the chemotherapeutical drug.	[93]
2016	Primary human ovarian cancer tissues and prostate cancer tissues	Microdissected cylindrical tissues	Two PDMS replicas with 5 open channels containing 5 microwells, fabricated by micromachined PMMA master molds, plasma-bonded	Drug treatment with carboplatin for 48 h	**On-chip:** Fluorescence staining and imaging of live tissues to assess cell viability **Off-chip:** Endpoint flow cytometry analysis to assess the survival of individual cells within the microtissues	The microfluidic platform was operated using simple instruments typically found in cell biology laboratories. Drug treatment response measured in the microfluidic chip was concordant with the clinical response of the patient.	[94]
2017	Primary human nasopharyngeal tumor tissues	Dissociated cells from tissues; 100–200 cells per droplet	PDMS microchannels containing 48 droplet formation wells, fabricated by a SU-8-patterned silicon wafer molds, plasma-bonded to glass coverslips	Drug treatment with bortezomib and cisplatin for 16–24 h	**On-chip:** Ethidium homodimer 1 labeling during cell seeding, brightfield and red fluorescence imaging to assess cell number and viability	The microfluidic system was capable of drug-screening as few as 16,000 cells obtained from primary cancer within 24 h after tumor resection from patients.	[95]
2018	Primary human mesothelioma tumor tissues	Dissociated cells from tissues mixed with hyaluronic acid and hydrogel precursor; 20 million cells/mL were seeded	Six chambers produced in an aluminum foil–adhesive film using a cutting plotter, attached to a glass slide (bottom) and polystyrene slide (top)	Drug treatment with carboplatin–pemetrexed or cisplatin–pemetrexed for 7 d	**On-chip:** Live–dead staining and fluorescence imaging to assess cell viability, proliferation assays, visualization of biomarkers using IHC *	The microfluidic platform was capable of maintaining the cell viability over 14 d and key mesothelioma biomarkers in patient-derived organoids (accurate tumor phenotype). Drug response of organoids was concordant with clinical outcomes. Patient-to-patient tumor heterogeneity was demonstrated.	[90]
2018	Primary human prostate cancer biopsies	Biopsies were minced, passaged and injected into the device; 24,000 cells were seeded	Two PDMS parts containing 240 square microwells fabricated by SU-8-patterned silicon wafer molds, plasma-bonded	Drug treatment with cisplatin, docetaxel and enzalutamide for 12 h	**On-chip:** Live–dead staining and fluorescence imaging to assess cell viability, calcein assay to assess concentration gradient formation **Off-chip:** RT-qPCR to assess prostate cancer cell gene expression	Proof of concept study. The microfluidic platform was capable of forming concentration gradient and maintain its stability for 12–16 h.	[96]
2018	Primary human triple-negative breast cancer tumor biopsy	1×10^5^ cells/mL were seeded; ~300 spheroids on the device	Two PDMS plasma-bonded layers with pillar array fabricated by two printed transparent film masks	Drug treatment with doxorubicin or docetaxel for 72 h	**On chip:** Live–dead staining assay, fluorescence imaging to assess spheroid number and size **Off-chip:** qRT-PCR to assess cancer stem cell marker expression	Proof of concept study. The microfluidic platform was capable of controlling the spheroid size. Spheroids showed a similar differential drug response observed in the patient.	[78]
2018	Primary human triple-negative breast cancer tumor tissue biopsies	Patient-derived tumor organoids (PDTO) from sectioned tissues; 1×10^7^ cells/mL cell suspension with Matrigel	PDMS device with 8 tumor tissue chambers fabricated by SU-8-patterned silicon wafer molds, plasma-bonded to a flat PDMS sheet	Drug treatment with paclitaxel for 48 h	**On-chip:** Immunostaining and fluorescence imaging to assess tumor growth, fluorescently tagged dextran perfusion via device to assess vessel permeability assessment	Proof of concept study. The microfluidic platform was capable of maintaining the viability of the primary tissue for up to 21 d. A tumor-on-a-chip device that mimics biological mass transport was designed, where 3D microvascular network was created prior to loading PDTO.	[97]
2018	Primary human glioblastoma tumor tissues	Dissociated cells from tissues; 0.5×10^6^ cells/mL were seeded	Poly-(ethylene glycol) diacrylate (PEGDA) hydrogel layer consisting of 7 channels with 9–11 microwells per channel, fabricated by printed plastic photomasks, crosslinked between two cover glass slides	Drug treatment with combination of bevacizumab and temozolomidefor 7 d	**On-chip:** Immunostaining and fluorescence imaging to assess spheroid formation **Off-chip:** Trypan blue staining to assess cell viability	Proof of concept study. Patient-to-patient tumor heterogeneity was demonstrated.	[88]
2019	Primary human small-cell lung cancer (SCLC) biopsies	Mechanically dissociated lung cancer organoids (LCOs) mixed with Matrigel	PDMS device consisting of 29 microwells fabricated by SU-8-patterned silicon wafer molds, plasma-bonded to a cover glass	Drug treatment with cisplatin and etoposide for 72 h	**On-chip:** Fluorescence imaging to assess organoid size, end point live–dead staining and fluorescence imaging to assess cell viability, apoptosis analysis **Off-chip:** Genomic analysis to evaluate the somatic mutations, qRT-PCR to characterize the specific marker expressions for cancer stem cells	First demonstration of 3D lung cancer organoid production from small-cell lung cancer tumors. The microfluidic device was capable of culturing these organoids, as well as performing drug sensitivity tests. The centers of the organoids could survive chemotherapy-induced cell death, which may help to elucidate chemotherapy resistance mechanisms.	[89]
2020	Primary metastatic human rectal tumor tissues	Tissue slices	PMMA plate consisting of 40 wells with an integrated channel network layer, fabricated by CO_2_ laser micromachining, sealed with chloroform vapor	Drug treatment with combinations of FOLFOX, FOLFIRI ** and staurosporine for 48 h	**Off-chip:** Proliferation assay and live–dead staining to assess cell viability, fluorescence imaging to assess cell death	The microfluidic device was capable of delivering multiplexed anti-cancer drugs on tumor slices and was compatible with on-chip live–dead staining.	[21]
2020	Primary human pancreatic ductal adenocarcinoma tumor organoid	Organoids were suspended in single cells and cultured in Matrigel with human dermal fibroblasts, before introducing this suspension (6800–13,600 cells) in HUVEC (75,000–12,5000 cells) scaffold	Two PDMS molds fabricated by SU-8-patterned silicon wafer molds, between PDMS molds and a PDMS sheet, pressed and perfused with a highly elastic polyester material, plasma-bonded to a silicon wafer	Drug treatment with gemcitabine for 96 h	**On-chip:** Luminescence assay to assess cell viability, fluorescence imaging to assess organoid size and morphology	Tumor-derived cells cultured in the microfluidic system only underwent ECM remodeling when co-cultured with fibroblasts. These changes, as well as vascularization, decreased the efficacy of gemcitabine treatment.	[98]
	**Radiotherapy**						
2019	Primary human head and neck squamous cell carcinoma tumor tissue	Tumor slices	Two PEEK plate parts consisting of 1 well for 1 sample, reversibly sealed by screws	Irradiation with a photon beam; 10 Gy in 5 × 2 Gy fractions in a 72 h schema; drug treatment with cisplatin for final 48 h alongside the 5 × 2 Gy irradiation fractions	**Off-chip:** Trypan blue/PI staining to assess tissue viability, flow cytometry to assess cell death, IHC * staining to assess radiation response markers	The microfluidic system was capable of maintaining the viability of precision-cut tumor slices for 68 h. This system enabled monitoring of the effects of irradiation and chemoradiation on tumor slices.	[15]
	**Immunotherapy**						
2018	Primary human non-small-cell lung cancer biopsies	Tumor fragments	Cyclic olefin copolymer (COC) device consisting of chevron-like 12-lane channel pattern, fabricated by micromachined aluminum master mold, bonded to a COC film using a heated lamination process	Treatment with an anti-PD-1 antibody pretreated tumor-infiltrating lymphocytes (TILs), monitored daily for 7–10 d	**On-chip:** Fluorescence staining and imaging to assess cell growth and viability **Off-chip:** Magnetic cell sorting using flow cytometry to separate tumor cells	The microfluidic system was capable of studying interactions between autologous lymphocytes and biopsy sample in response to an anti-PD-1 antibody. The sample showed responder behavior, mimicking the in vivo tumor response.	[72]
2018	Primary human metastatic melanoma tumor tissues	Minced tumor tissues mixed with collagen	Cyclic olefin polymer device by AIM BIOTECH consisting of 3 chambers	Treatment with anti-PD-1 (pembrolizumab, 250 μg/mL), anti-CTLA-4 (ipilimumab, 50 μg/mL) or combination for 5–9 d	**Oh-chip:** Live–dead staining and fluorescence imaging to assess cell viability **Off-chip:** Flow cytometry for immune profiling, RNA-seq, cytokine profiling in media	The microfluidic system was capable of performing a range of on-/off-chip analyses. It demonstrated the immune checkpoint sensitivity of patient-derived tumor spheroids, which is not seen in 2D cultures.	[92]
2019	Primary human non-small-cell lung carcinoma tumor biopsies	Tumor fragments	Pro3dure GR-10 resin device fabricated by digital light projection stereolithography (DLP-SLA) 3D printing	Treatment with anti-PD-1 antibodies, monitored after 24, 48 and 72 h	**On-chip:** Live–dead staining and fluorescence imaging to assess cell viability, resident lymphocyte response to selected antibodies **Off-chip:** Fluorescence imaging to determine healthy tumor fragments	The microfluidic system was capable of culturing biopsied tumor tissue and resident lymphocytes in a dynamic perfusion system. It enabled the monitoring of tumor response to immunotherapeutic agents. Clinical correlation of the laboratory results is needed to determine its utility in guiding personalized medicine approaches.	[17]
	**Combined therapy**						
2018	Primary human pancreatic tumor biopsies	Dissociated cells from tumors; ~100 cells encapsulated in each plug	Droplet-based PDMS device consisting of 1140 plugs, fabricated by AZ-40XT patterned silicon wafer molds, plasma-bonded to a thin elastic PDMS membrane with integrated Braille valves	Drug treatment with chemotherapeutic drugs (oxaliplatin and gemcitabine), specific kinase targets (Cyt387, PHT-427, MK-2206, GDC0941, gefitinib, ACHP, AZD6244) and one cytokine (tumor necrosis factor-α) for up to 14 d	**On-chip:** Fluorescence staining and imaging to assess cell viability, caspase-3 activity to access cell apoptosis	The microfluidic system was capable of screening 62 different drug conditions on biopsy-derived cells. No drug combination had strong efficacy across all patient samples, encouraging the consideration of personalized medicine approaches to pancreatic cancer	[14]

* IHC = immunohistochemistry staining; ** FOLFOX = 5-FU and Oxaliplatin; FOLFIRI = 5-FU and Irinotecan.

## Data Availability

Not applicable.
